# Efficacy and Prognostic Indicators of Isatuximab, Pomalidomide, and Dexamethasone (IsaPd) in Daratumumab‐Refractory Multiple Myeloma Patients: A Multicenter Real‐World Study

**DOI:** 10.1002/hon.70042

**Published:** 2025-02-03

**Authors:** Enrica Antonia Martino, Daniele Derudas, Elena Rossi, Paola Stefanoni, Silvia Mangiacavalli, Elena Zamagni, Massimo Offidani, Anna Furlan, Angela Maria Quinto, Roberta Della Pepa, Giuseppe Bertuglia, Emiliano Barbieri, Concetta Conticello, Claudio De Magistris, Velia Bongarzoni, Anna Maria Cafro, Anna Mele, Cirino Botta, Nicola Sgherza, Giuseppe Mele, Ombretta Annibali, Angela Rago, Raffaele Fontana, Ernesto Vigna, Antonella Bruzzese, Katia Mancuso, Angela Amendola, Annalisa Citro, Emilia Cotzia, Sonia Morè, Elena Rivolti, Loredana Pettine, Monica Galli, Valerio De Stefano, Maria Teresa Petrucci, Alessandro Corso, Antonino Neri, Francesco Di Raimondo, Niccolò Bolli, Pellegrino Musto, Fortunato Morabito, Massimo Gentile

**Affiliations:** ^1^ Department of Onco‐hematology Hematology Unit Azienda Ospedaliera Annunziata Cosenza Italy; ^2^ Department of Hematology Businco Hospital Cagliari Italy; ^3^ Section of Hematology Catholic University Fondazione Policlinico Gemelli IRCCS Rome Italy; ^4^ Hematology and Bone Marrow Transplant Unit Bergamo Italy; ^5^ Division of Hematology IRCCS Fondazione Policlinico San Matteo Pavia Italy; ^6^ IRCCS Azienda Ospedaliero‐Universitaria di Bologna Istituto di Ematologia “Seràgnoli” Bologna Italy; ^7^ Dipartimento di Scienze Mediche e Chirurgiche Università di Bologna Bologna Italy; ^8^ Hematology Unit AOU delle Marche Ancona Italy; ^9^ Division of Hematology Ospedale Ca' Foncello di Treviso Treviso Italy; ^10^ Haematology and Transplant Unit IRCCS ‐ Istituto Tumori “Giovanni Paolo II” Bari Italy; ^11^ Department of Clinical Medicine and Surgery University Federico II Naples Italy; ^12^ Division of Hematology Azienda Ospedaliero‐Universitaria Città della Salute e della Scienza di Torino University of Torino Torino Italy; ^13^ Hematology Unit Azienda USL‐IRCCS di Reggio Emilia Reggio Emilia Italy; ^14^ Division of Hematology Azienda Policlinico‐S. Marco University of Catania Catania Italy; ^15^ Hematology Unit Fondazione IRCCS Ca' Granda Ospedale Maggiore Policlinico Milan Italy; ^16^ UOC of Hematology San Giovanni‐Addolorata Hospital Rome Italy Rome Italy; ^17^ Department of Hematology GOM Niguarda Hospital Milan Italy; ^18^ Haematology Ospedale Cardinale Panico Tricase Italy; ^19^ Department of Health Promotion Mother and Child Care Internal Medicine and Medical Specialties University of Palermo Palermo Italy; ^20^ Unit of Hematology and Stem Cell Transplantation AOUC Policlinico Bari Italy; ^21^ Department of Hematology Hospital Perrino Brindisi Italy; ^22^ Hematology stem cell transplantation Fondazione Policlinico Universitario Campus Bio medico di Roma Rome Italy; ^23^ UOSD Ematologia ASL Roma 1 Rome Italy; ^24^ Hematology and Transplant Center University Hospital “San Giovanni di Dio e Ruggi d'Aragona” Salerno Italy; ^25^ Hematology Unit Azienda Ospedaliera Regionale “San Carlo” Potenza Italy; ^26^ Hematology Unit Legnano General Hospital Legnano Italy; ^27^ Section of Hematology‐ Ospedale E. Muscatello‐Augusta Siracusa Italy; ^28^ Scientific Directorate Azienda USL‐IRCCS di Reggio Emilia Emilia Italy; ^29^ Department of Precision and Regenerative Medicine and Ionian Area “Aldo Moro” University School of Medicine Bari Italy; ^30^ Gruppo Amici Dell'Ematologia Foundation‐GrADE Reggio Emilia Italy; ^31^ Department of Pharmacy Health and Nutritional Science University of Calabria Rende Italy

**Keywords:** dara‐refractory, dexamethasone, isatuximab, multiple myeloma, pomalidomide, salvage therapy

## Abstract

This multicenter real‐world analysis evaluated the efficacy of isatuximab, pomalidomide, and dexamethasone (IsaPd) in 51 patients with multiple myeloma (MM) who were refractory to daratumumab (Dara‐R). The majority were under 70 years old (60.8%), predominantly female (56.9%), and heavily pretreated, with 74.5% being triple‐class refractory (TCR); 32.1% of the 28 patients with cytogenetic data had high‐risk abnormalities. The overall response rate (ORR) was 56.9%, including 3 patients with stringent complete response (sCR), 4 with CR, and 7 with very good partial response (VGPR). Neither age, number of prior therapies, TCR status, nor time from Dara refractoriness to IsaPd initiation significantly affected response rates.

Median progression‐free survival (PFS) was 5.8 months, with a 12‐month PFS probability of 30.6%. Baseline hemoglobin (Hb) levels were a key predictor of PFS: patients with Hb < 11.8 g/L had a 3.5‐fold increased risk of progression, with a median PFS of 4.6 months compared to 22 months in those with higher Hb.

Median overall survival (OS) was 21.0 months, with a 12‐month OS probability of 63.4%. Lower Hb levels (< 11 g/L) were associated with a tenfold increased risk of mortality.

Among the 28 patients who underwent FISH analysis, while no significant difference in mortality risk was observed, those with high‐risk cytogenetic abnormalities exhibited a nearly tenfold increased risk of disease progression.

These results suggest that IsaPd offers a meaningful option for Dara‐R patients, with Hb levels serving as a critical predictor of both PFS and OS. However, PFS remains modest, underscoring the need for novel combination therapies.

## 1 Introduction

Despite recent advances in multiple myeloma (MM) treatment, most patients eventually develop resistance to available therapies. This resistance leads to progressively shorter remission periods and a decline in median overall survival (OS), especially in heavily pre‐treated individuals [1]. Daratumumab (Dara), a monoclonal antibody (mAb) targeting CD38, has become a cornerstone in the treatment of MM, particularly in combination with immunomodulatory agents, proteasome inhibitors, and corticosteroids [2]. However, as the use of Dara has increased in earlier lines of therapy, the number of patients who become refractory to this agent has grown significantly. Dara‐refractory (Dara‐R) MM is associated with a particularly poor prognosis, as these patients have often developed resistance to multiple drug classes, including immunomodulatory drugs and proteasome inhibitors [3]. Once patients become refractory to Dara, treatment options are limited, and outcomes typically worsen, with progression‐free survival (PFS) and OS substantially reduced compared to earlier stages of the disease [1, 2, 3].

The therapeutic landscape for Dara‐R MM is rapidly evolving, addressing the challenges of managing this difficult‐to‐treat population, as highlighted by the MAMMOTH study [3]. In this patient subset, advanced therapeutic options, including B‐cell maturation antigen (BCMA)‐targeted CAR‐T therapies such as idecabtagene vicleucel (ide‐cel) and ciltacabtagene autoleucel (cilta‐cel), as well as bispecific antibodies like teclistamab, have shown remarkable efficacy, achieving high response rates and prolonged remissions. Ide‐cel and cilta‐cel have demonstrated exceptionally high ORRs ranging from 73% to 97%. In the pivotal Phase 1/2 MajesTEC‐1, teclistamab achieved an ORR of 63% with a median PFS of 11.3 months among 165 relapsed or refractory MM patients, most of whom were triple class refractory (TCR) [4]. Selinexor‐based regimens demonstrate promise in Dara‐R patients, but their long‐term benefit remains to be fully established [5]. Moreover, a Phase II study formally assessed elotuzumab associated with pomalidomide and dexamethasone (EloPd) in Dara‐R MM patients [6], also supported by real‐world data [7, 8, 9] poses this regimen as another option.

The rationale for using isatuximab (Isa) in patients Dara‐R is driven by both the differences in their mechanisms of action [10, 11] and promising clinical results, particularly in heavily pretreated patients [12, 13, 14, 15, 16, 17]. Isa's unique binding to a distinct epitope on the CD38 molecule compared to Dara is one of the critical differences that may enable it to retain efficacy even in Dara‐R patients [11]. Dara is primarily known for mediating its effects through immune effector functions like antibody‐dependent cellular cytotoxicity (ADCC), antibody‐dependent cellular phagocytosis (ADCP), and complement‐dependent cytotoxicity (CDC) [10, 11]. On the other hand, Isa not only induces these immune‐mediated responses but also has direct cytotoxic effects on MM cells. Specifically, Isa triggers apoptosis without the need for Fc receptor engagement or cross‐linking, a feature not shared by Dara [18]. This ability to directly kill MM cells independent of immune effector cells is particularly valuable in cases where the patient's immune system may be compromised or dysfunctional, as is common in advanced MM [10]. Furthermore, Isa modulates CD38 enzymatic activity, impacting intracellular signaling pathways that regulate tumor cell survival, such as the NAD + metabolism and calcium signaling, which are crucial for MM cell growth [10, 11]. This difference may allow Isa to overcome resistance mechanisms that impair Dara's efficacy, such as CD38 downregulation and impaired ADCC and CDC [10, 11]. Additionally, Dara resistance can involve immune evasion strategies, including the release of CD38‐expressing microvesicles, which Isa might bypass due to its distinct apoptotic pathways. Moreover, Isa's ability to enhance natural killer (NK) cell and T‐cell activity points to its potential to restore or augment immune‐mediated responses that may have been diminished by prior Dara therapy [19, 20]. Therefore, the combination of these distinct biological properties and encouraging clinical data provides a strong rationale for the use of Isa in patients who have progressed on or become refractory to Dara.

Limited clinical data currently support the rationale for using Isa in Dara‐R patients [21, 22, 23]. Key trials such as ICARIA‐MM [14, 15] and IKEMA [17], have investigated the efficacy of Isa in combination with pomalidomide and dexamethasone (IsaPd), and with carfilzomib and dexamethasone (IsaKd), respectively, demonstrating significant improvements in ORR, PFS and OS in RRMM patients with these triplet regimens. However, these studies each included only one patient who had received Dara in a prior line of therapy, limiting specific insights into Isa's efficacy in the Dara‐R setting.

In a recent study, we contributed significant real‐world evidence on the efficacy and safety of the IsaPd regimen in a cohort of 270 RRMM patients across Italian centers [21]. Within this cohort, 50 patients were identified as refractory to prior Dara treatment. The inclusion of an additional case with comparable characteristics allowed for a comprehensive analysis, facilitating an important evaluation of IsaPd efficacy specifically in this challenging subgroup. Herein, we aim to highlight and further analyze the outcomes in this Dara‐R population.

## 2 Methods

### 2.1 Patients

This retrospective analysis was conducted on a cohort of RRMM patients treated with IsaPd across 51 centers in Italy. Clinical data were extracted from medical records and compiled into a centralized database containing clinical information such as age, gender, date of diagnosis, laboratory parameters, treatment history, and date of last follow‐up or death. Data collection began at the time of inclusion and was updated on an ongoing basis. The study cohort consisted of 51 consecutive patients with RRMM who received at least one cycle of IsaPd as salvage treatment between January 2021 and June 2024. TCR patients were defined as those who were refractory to an anti‐CD38 monoclonal antibody, a proteasome inhibitor (PI), and an immunomodulatory drug (IMiD). All patients were treated with IsaPd according to marketing approval guidelines, as previously described [14]. Specifically, Isa was administered intravenously at a dose of 10 mg/kg on days 1, 8, 15, and 22 during the first cycle, followed by administration on days 1 and 15 of subsequent cycles. Pomalidomide was given orally at 4 mg once daily on days 1–21 of each cycle, and dexamethasone at the dose of 40 mg (or 20 mg in patients over 75 years of age) once weekly. Premedication included diphenhydramine (25–50 mg) or its equivalent, ranitidine (50 mg) or its equivalent, and acetaminophen (650–1000 mg) or its equivalent, administered 30–90 min before Isa infusion. Dexamethasone was administered prior to Isa infusion as part of both premedication and study treatment.

According to each center's policy, during treatment, all patients received prophylactic antibacterial, antiviral, and antithrombotic therapy. IsaPd was continued in 28‐day cycles until disease progression, unacceptable toxicity, or withdrawal of consent.

Time‐to‐event endpoints evaluated in this study included PFS and OS. Treatment response and disease progression were evaluated according to the International Myeloma Working Group (IMWG) criteria [22, 23], with response defined as achieving at least a partial remission (PR). Refractory myeloma is defined as a disease that does not respond to primary or salvage therapy or progresses within 60 days of the last therapy. Biochemical relapse was characterized by an increase in serum and/or urine monoclonal protein alone, whereas clinical relapse referred to relapse accompanied by progression‐related features, including CRAB symptoms, that is, hypercalcemia (C), renal failure (R), anemia (A), and bone disease (B). Finally, high‐risk cytogenetic risk was defined as the presence of at least one of the following: t (4; 14), t (14; 16), and del (17p) [1].

The study protocol was approved by the Ethics Committees of each participating institution in accordance with the principles of the Declaration of Helsinki.

The study protocol was reviewed and approved by the Institutional Ethics Committees in accordance with the principles of the Declaration of Helsinki.

### 2.2 Statistical Analysis

Categorical variables were compared using two‐way tables with Fisher's exact test and multi‐way tables with Pearson's Chi‐square test. Multivariable ordinal regression analysis was employed to assess the influence of potential confounders on the association between the best response and variables found to be statistically significant on univariable analysis by Pearson chi‐square or Fisher's exact test.

The Kaplan‐Meier method was used to analyze PFS, and OS, measured from the initiation of RRMM IsaPd treatment until respectively death from any cause or progression or last follow‐up, the earliest start date of subsequent therapy or last follow‐up, and death from any cause or last follow‐up. The predictive cut‐off value of Hb levels (optimal threshold as identified by the Youden index) for discriminating patients who progressed or died or died for any cause from those without these outcomes was identified by the Receiver Operating Characteristic (ROC) curve analysis. The optimal cut‐off corresponds to the Hb threshold that maximizes the difference between true positives (sensitivity) and false positives (1‐specificity) for predicting the occurrence of these outcomes.

The statistical significance of associations between individual variables and survival outcomes was calculated using the log‐rank test. The prognostic impact of the outcome variable was further investigated by univariable and multivariable Cox regression analysis, with results expressed as hazard ratios (HR) and 95% confidence intervals (CI). A value of *p* ≤ 0.05 was considered statistically significant. Data analysis was performed by STATA for Windows v.9 and SPSS Statistics v.21.

## 3 Results

### 3.1 Patient Characteristics

This retrospective cohort undergoing treatment with IsaPd included 51 patients. The majority of patients were younger than 70 years (60.8%), with a slight predominance of female patients (56.9%). IgG was the predominant paraprotein subtype (66.7%), with smaller proportions exhibiting IgA (21.6%) or light‐chain‐only disease (11.7%) (Table 1). Renal function was preserved in 70.6% of patients (CrCl ≥ 60 mL/min), while 29.4% exhibited renal impairment. Disease staging, according to the International Staging System (ISS), was evenly distributed across stages I (41.2%) and II (43.1%), with 15.7% of patients in stage III. The median Hb level was 11.1 g/L, and elevated lactate dehydrogenase (LDH) levels were observed in 19.6% of the cohort. Patients were heavily pretreated, with 37.3% receiving two prior lines of therapy, 33.3% receiving three, and 29.4% receiving four or more. Additionally, 58.8% had previously undergone autologous stem cell transplantation (ASCT) (Table 1). All patients were refractory to both Dara and lenalidomide, with 74.5% also refractory to proteasome inhibitors, classifying nearly three‐quarters of the cohort as TCR. The median number of cycles of Dara‐containing regimens received was 10 (range 1–36). The median interval between the discontinuation of Dara therapy and the initiation of IsaPd treatment was 10 months (range 1–41 months). Dara‐based regimens consisted of Dara‐lenalidomide‐dexamethasone (22 patients), Dara‐bortezomib‐dexamethasone (10 patients), Dara single agent (7 patients), Dara‐bortezomib‐melphalan‐dexamethasone (5 patients), Dara‐bortezomib‐thalidomide‐dexamethasone (5 patients), Dara‐bortezomib‐cyclofosphamide‐dexamethasone (1 patient), and Dara‐bortezomib‐lenalidomide‐dexamethasone (1 patient). At the initiation of IsaPd therapy, the majority of patients presented with either symptomatic relapse (39.2%) or refractory disease (43.1%), with 17.6% experiencing biochemical relapse. Cytogenetic analysis, available for 28 patients, revealed 32.1% classified as high‐risk, due to aberrations such as t (4; 14), t (14; 16), and del (17p) (Table 1).

**TABLE 1:** Main characteristics of patients at isatuximab, pomalidomide, and dexamethasone initiation.

	No. of patients (%)
**Age, (years) median (range)**	67 (48–81)
< 70	31 (60.8)
≥ 70	20 (39.2)
**Gender**	
Male	22 (43.1)
Female	29 (56.9)
**Paraproteins (isotype**)	
Immunoglobulin G	34 (66.7)
Immunoglobulin A	11 (21.6)
Light chain only	6 (11.7)
**Creatinine clearance (mL/min) median (range)**	70 (9–157)
≥ 60	36 (70.6)
< 60	15 (29.4)
**International staging system**	
I	21 (41.2)
II	22 (43.1)
III	8 (15.7)
**Hb gr/L, median (IQR)**	11.1 (9.8–12.3)
**LDH**	
Normal	41 (80.4)
Elevated	10 (19.6)
**Previous lines of therapy**	
2	19 (37.3)
3	17 (33.3)
≥ 4	15 (29.4)
**Previous autologous stem cell transplantation**	
No	21 (41.2)
Yes	30 (58.8)
**Daratumumab refractory**	
Yes	51 (100)
No	0 (0)
**Daratumumab‐IsaPD interval, median time (IQR)**	10 (5–20)
**Daratumumab as the last therapy**	
Yes	12 (23.5)
No	39 (76.5)
**Lenalidomide refractory**	
Yes	51 (100)
No	0 (0)
**Proteasome inhibitor refractory**	
Yes	38 (74.5)
No	13 (25.5)
**Disease status**	
Biochemical relapse	9 (17.6)
Symptomatic relapse	20 (39.2)
Refractory disease	22 (43.1)
**Cytogenetic analysis (n = 28)**	
Standard risk	19 (67.9)
High risk	9 (32.1)

### 3.2 Response Evaluation

At the last follow‐up, all 51 patients were evaluable for response. A total of 29 patients (56.9%) reached at least a PR. Specifically, 3 patients achieved a stringent complete response (sCR), 4 a complete response (CR), 7 a very good partial response (VGPR), and 15 a PR. Age (< 70 years vs. ≥ 70 years), gender, number of prior lines of therapy (2 vs. > 2), previous ASCT, MM status at IsaPd initiation (i.e., biochemical relapse vs. symptomatic relapse vs. refractory to last treatment), CrCl, ISS, LDH as well as the TCR status did not significantly impact the likelihood of achieving at least a PR to IsaPd (data not shown).

Likewise, the interval between Dara refractoriness and IsaPd initiation (i.e., 6 months, 12 months), as well as whether patients received IsaPd immediately following a Dara‐containing regimen or after other intervening therapies did not appear to significantly impact ORR (Table 2). However, patients initiating IsaPd more than 12 months after Dara treatment showed a higher ORR compared to those with an interval of less than 12 months (70.8% vs. 44.4%), with this difference approaching statistical significance (*p* = 0.06) (Table 2).

**TABLE 2:** Impact of Dara‐containing regimen timing on clinical outcomes.

Features	ORR (%)	*p*	12‐month PFS probability (%)	*p*	12‐month OS probability (%)	*p*
Interval time between Dara‐containing regimen and IsaPd (months)						
≥ 6 vs. < 6	62.9/43.8	0.2	33.1/24.7	0.35	69.5/51.4	0.88
≥ 12 vs. < 12	70.8/44.4	0.06	36.5/24.9	0.2	76/51.1	0.68
Lines of therapy between Dara‐containing regimen and IsaPd						
0 vs. ≥ 1	56.4/58.3	0.9	40.4/27.9	0.54	50.5/68.5	0.59

### 3.3 Progression‐Free Survival

**FIGURE 1:**
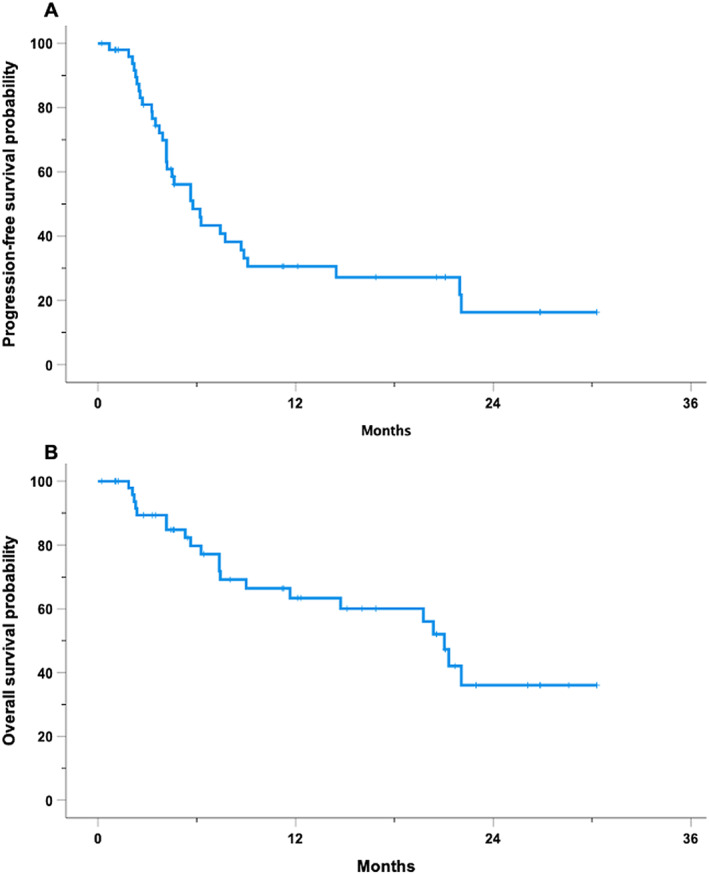
Kaplan Meier curves for 51 RRMM patients treated with Isatuximab, pomalidomide, and dexamethasone. **Panel A**. Kaplan Meier curve of PFS; **Panel B**. Kaplan Meier curve of OS.

**FIGURE 2:**
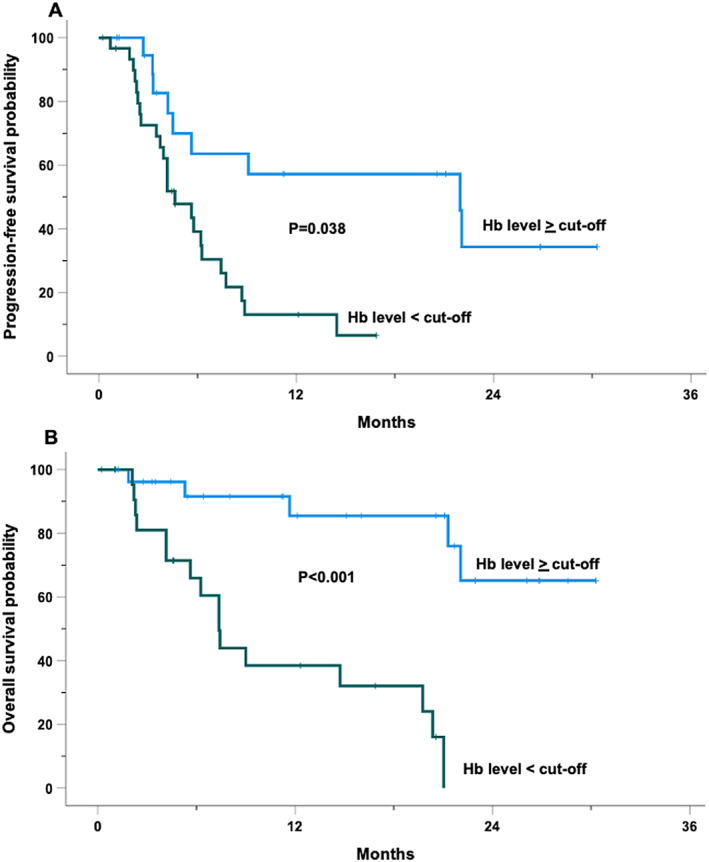
Kaplan Meier curves for 51 RRMM patients treated with Isatuximab, pomalidomide, and dexamethasone cistered by Hb cut‐off levels as determined by ROC analyses. **Panel A**. Kaplan Meier curve of PFS; **Panel B**. Kaplan Meier curve of OS.

At a median follow‐up of 16.8 months (IQR 10.5–23.2), 33 patients (64.7%) experienced disease progression or death. The median PFS was 5.8 months (95% CI, 3.9–7.7 months), with a 12‐month PFS probability of 30.6% (Figure 1A). At univariable analysis, none of the factors indicated in Figure S1A showed any statistically significant association with PFS, although age > 70 years and receiving more than two prior lines of therapy approached borderline significance. Notably, both the time from Dara discontinuation to IsaPd start (Table 2), as well as the number of Dara cycles, introduced as a continuous variable in a Cox univariable analysis, failed to demonstrate any significant association with the risk of progression (HR 0.98, 95% CI 0.95‐1.0, *p* = 0.2; HR 0.99, 95% CI 0.95‐1.05, *p* = 0.9, respectively). Additionally, patients who received IsaPd as a third‐line treatment demonstrated a longer PFS, with a trend toward statistical significance, compared to those treated with the regimen in later lines of therapy (HR 2, 95% CI 0.92‐4.47, *p* = 0.078). Conversely, patients with Hb < 11.8 gr/L, a value detected by ROC curve analysis (Figure S2A), were 3.5 times more likely to experience disease progression compared to those with higher Hb levels (HR 3.5, 95% CI 1.5–8.4). The median PFS for patients with Hb ≥ 11.8 g/dL was 22 months (95% CI, 6.2–37.7 months), compared to 4.6 months (95% CI, 3.0–6.3 months) for those with lower Hb levels (Figure 2A).

### 3.4 Overall Survival

The median OS for this cohort was 21.0 months (95% CI, 19.0–23.0 months), with a 12‐month OS probability of 63.4% (Figure 1B). None of the variables indicated in Figure S1B, analyzed in the univariable model showed a statistically significant association with OS. Again, both the interval time from Dara discontinuation for refractoriness to IsaPd start (Table 2), as well as the number of Dara cycles introduced as continuous variables in a Cox univariable analysis, failed to demonstrate any significant association with the risk of death (HR 0.98, 95% CI 0.96‐1.0, *p* = 0.4; HR 1.02, 95% CI 0.99‐1.06, *p* = 0.2, respectively). However, Hb < 11 g/L, as determined by ROC analysis (Figure S2B) emerged as a strong predictor of shorter OS, with patients exhibiting an approximately tenfold increased risk of mortality in the univariable analysis (HR 10.7, 95% CI 3.0–37.9) (Figure 2B).

As an ancillary observation, among the 28 cases available for FISH analysis, patients with high‐risk cytogenetic abnormalities exhibited a roughly tenfold increased risk of disease progression (HR 9.6, 95% CI 2.4‐38.3, *p* < 0.0001). Nevertheless, no significant difference in mortality risk was observed (HR 2.0, 95% CI 0.6‐6.5, *p* = 0.2).

## 4 Discussion

The therapeutic landscape for RRMM is evolving rapidly, and the management of Dara‐R and TCR patients represents a critical challenge [1, 2, 3]. The therapeutic options available for Dara‐R patients remain limited, and these patients often exhibit multidrug resistance, including refractoriness to lenalidomide and proteasome inhibitors, placing them in the difficult‐to‐treat TCR category [1, 2, 3]. Addressing this unmet need requires innovative approaches that leverage different mechanisms of action to overcome treatment resistance.

One of the key takeaways from these findings is that IsaPd offers a viable treatment option for patients who are refractory to Dara. Given that nearly 75% of the cohort was TCR, the response rate of 56.9% is noteworthy. This suggests that Isa, while also targeting CD38, may engage different mechanisms compared to Dara, potentially overcoming resistance in patients who have failed Dara‐based therapies [10, 11]. Such findings are encouraging in a landscape where treatment options for TCR patients are limited [1, 2, 3, 4, 5, 6, 7, 8, 9], highlighting IsaPd as a valuable alternative for those previously deemed unresponsive to CD38‐targeted therapy.

However, it is noteworthy that despite the encouraging response rates, the study's survival data offer additional context for understanding the utility of IsaPd in this patient population. With a median PFS of 5.8 months and a 12‐month PFS probability of 30.6%, it is clear that while IsaPd can induce responses, the durability of these responses remains modest. Several studies have investigated the efficacy of Isa in RRMM patients who were refractory to Dara. In a prospective Phase 2 study [24], 32 heavily pretreated RRMM Dara‐R patients, received Isa as monotherapy. Unlike our analysis, which demonstrated an ORR of nearly 57%, the Phase 2 study observed no objective responses; instead, only one patient achieved a minimal response (MR) and 17 exhibited stable disease (SD). Notably, in the SD subgroup, the longest duration of response was 18.5 months. Moreover, the disease control rate in the prospective study was superior among patients with a prolonged interval between the last Dara dose and the first Isa dose. The significance of the interval between the administration of these two anti‐CD38 monoclonal antibodies was further emphasized in a real‐world study [25].

In this study, a Japanese group analyzed the outcome of 37 patients who received Isa after developing refractoriness to Dara and found a median PFS of 5.1 months, consistent with outcomes in our cohort. Unlike our findings, Kikuchi et al. observed that an interval longer than 3 months between the last Dara dose and the first Isa dose correlated with longer PFS, although it did not influence OS.

Although patients receiving IsaPd more than 12 months after a Dara‐containing regimen exhibited a higher ORR, with a trend toward statistical significance, the interval between the two anti‐CD38 monoclonal antibody treatments did not appear to influence PFS and OS outcomes in our cohort.

Moreover, patients with elevated LDH levels and those with TCR had poorer outcomes, underscoring the challenges in achieving disease control within this subset. In a large‐scale French real‐world study, 294 RRMM patients treated with IsaPd were analyzed, including 56 patients who were classified as Dara‐R. This Dara‐R cohort exhibited a shorter median PFS compared to patients previously exposed to, but not refractory to, anti‐CD38 monoclonal antibodies or those who were anti‐CD38 naïve (3 months vs. 9.5 and 16.6 months, respectively) [26].

Given these findings, along with studies demonstrating the benefit of retreatment strategies with novel molecules for sustained disease control [27, 28], there has been growing interest in re‐evaluating Dara retreatment efficacy. Small retrospective analyses have reported modest ORR with Dara retreatment [29, 30]. Nevertheless, recent in vitro analysis suggested that Dara retreatment could be a viable option for patients who have been off treatment for at least 12 months, while switching to an alternative anti‐CD38 agent like Isa may yield superior outcomes in cases of shorter intervals [31].

In the last decades, other therapeutic strategies, such as selinexor‐based regimens [5], offer the potential for Dara‐R patients, particularly due to selinexor's novel mechanism of action as a selective inhibitor of nuclear export. However, while selinexor has demonstrated promise, its long‐term benefit and tolerability remain areas of active investigation. Similarly, regimens incorporating elotuzumab, another monoclonal antibody with a distinct target (SLAMF7), have shown moderate efficacy in Dara‐R patients, as seen in the pivotal Phase II trial [6] and real‐world settings [7, 8, 9]. The combination of EloPd has shown encouraging outcomes, but its role in overcoming CD38 refractoriness is less well‐defined compared to Isa.

A closer examination of specific factors influencing PFS yields a compelling observation: baseline Hb levels significantly impact PFS. Patients with Hb levels below 11.8 g/L were 3.5 times more likely to experience disease progression compared to those with higher levels, and this disparity was reflected in OS as well. Those with Hb below 11 g/L exhibited an approximately tenfold increased risk of mortality, underlining the prognostic value of Hb in this setting.

In comparing the efficacy of IsaPd and EloPd regimens, the observed outcomes highlight both shared challenges and distinct features of these treatment options for Dara‐R patients belonging to our real‐world cohorts [7, 8, 9, 21]. Specifically, in the EloPd group we identified ISS stages II and III, low Hb levels, the last therapy being Dara, and symptomatic relapse as significant predictors of shorter PFS in multivariable analysis. Advanced ISS stages, low Hb levels, symptomatic relapse, and refractory disease also negatively impacted OS [7, 8, 9]. Notably, low Hb levels, albeit at different cut‐offs, were identified in both treatment groups as negative prognostic indicators of patient outcomes, serving as a surrogate marker for more aggressive disease biology or diminished bone marrow reserve. Nevertheless, a comparative analysis between EloPd and IsaPd revealed no significant differences in PFS or OS within our real‐world cohorts, even when the multivariate analysis was adjusted for hemoglobin levels (data not shown).

As an ancillary finding, we found that cytogenetic abnormalities were also predictive of disease progression, with high‐risk cytogenetics conferring a nearly tenfold increased risk of progression. Conversely, high‐risk cytogenetics did not significantly impact OS in our study. Notably, cytogenetic data were available for only approximately half of the patients, highlighting the need for validation in a larger cohort to confirm the robustness of these findings. However, it is plausible that cumulative resistance mechanisms arising from multiple lines of treatment may reduce the prognostic relevance of conventional cytogenetic markers. This underscores the potential utility of more comprehensive biological indicators—such as those derived from liquid biopsy [32]—which may offer more reliable insights into treatment response and survival outcomes. Such broad‐spectrum biomarkers could be especially valuable in Dara‐R patients, in whom traditional cytogenetic influences on prognosis may be increasingly offset by accumulated resistance mechanisms.

Isa's distinct mechanism of action, which includes direct cytotoxic effects independent of immune effector cells, as well as modulation of CD38 enzymatic activity, likely contributes to its ability to overcome resistance mechanisms associated with Dara failure [10, 11]. This may explain why IsaPd remains effective in a significant proportion of Dara‐R patients.

However, the relatively short median PFS observed in our cohort highlights the need for novel combinations or sequencing strategies that can further enhance the durability of responses in this refractory population. CAR‐T therapies targeting BCMA, such as ide‐cel and autoleucel cilta‐cel, have demonstrated high response rates and durable remissions even in heavily pretreated populations [4]. These therapies work by harnessing the patient's T‐cells to recognize and destroy MM cells, offering a potent and individualized treatment option. However, access to CAR‐T is limited by logistical challenges, manufacturing time, and patient eligibility due to advanced disease or poor performance status. Bispecific antibodies, such as teclistamab [33], as well as antibody‐drug conjugates, such as belantamab mafodotin [34], represent an exciting alternative, offering either off‐the‐shelf options that can engage the immune system to target BCMA without the need for personalized T‐cell engineering or deliver cytotoxic drugs specifically in MM cells. These agents have shown efficacy in TCR and Dara‐R patients, providing a new therapeutic avenue for those with limited options. While both CAR‐T and bispecific antibodies are highly promising, they also present challenges, including the potential for immune‐related toxicities, such as cytokine release syndrome, and the durability of responses in the absence of continued treatment. A critical area of ongoing research is identifying the optimal sequencing of these therapies.

In conclusion, this study represents, to our knowledge, the largest real‐world investigation into the outcomes of Dara‐R patients treated with Isa. Our findings highlight Hb levels serve as a crucial predictor of clinical outcomes in this population. This biomarker, alongside emerging immune‐based therapeutic approaches, may play a pivotal role in refining treatment strategies and optimizing outcomes for Dara‐R RRMM patients.

## Author Contributions

E.A.M, M.G., F.D.R., V.D.S., N.B., A.C., A.N., F.M., and P.M. designed the study; M.G. and F.M. performed statistical analysis; D.D., E.R., P.S., J.M., S.M., E.Z., M.O., A.F., A.M.Q., R.D.P., G.B., E.B., C.C., C.D.M., V.B., A.M.C., A.M., C.B., N.S., G.M., O.A., A.R., R.F., E.V., A.B., K.M., A.A., A.Ci., E.C., S.M., E.R., L.P., M.Ga., M.T.P., analyzed and interpreted data. E.A.M., M.G., and F.M. wrote the manuscript; all authors gave final approval.

## Ethics Statement

The study protocol was reviewed and approved by the Institutional Ethics Committees in accordancewith the principles of the Declaration of Helsinki.

## Conflicts of Interest

The authors declare no conflicts of interest..

### Peer Review

The peer review history for this article is available at https://www.webofscience.com/api/gateway/wos/peer-review/10.1002/hon.70042.

## Supporting information

Supporting Information S1

Figure S1A

Figure S1B

Figure S2A

Figure S2B

## Data Availability

The data that support the findings of this study are available from the corresponding author upon reasonable request.
