# Supplementary material for: Efficacy and Prognostic Indicators of Isatuximab, Pomalidomide, and Dexamethasone (IsaPd) in Daratumumab‐Refractory Multiple Myeloma Patients: A Multicenter Real‐World Study
Source: Hematol Oncol. 2025 Feb 3;43(2):e70042. doi: 10.1002/hon.70042 (PMC11789454; doi:10.1002/hon.70042)
Supplement: Supplementary file 1 — Supporting Information S1 [file HON-43-e70042-s003.docx]

Supplementary Figure 1. Forest plot of Cox univariable analysis for (Panel A) progression-free survival and (Panel B) overall survival, by

12 potential predictive variables.

Supplementary Figure 2. Receiver Operating Characteristic (ROC) analysis of Hb level to identify patients who progressed (Panel A), and patients who died (Panel B). The dashed line represents the reference line of prognostic usefulness.
