# Supplementary figures and images for: Efficacy and Prognostic Indicators of Isatuximab, Pomalidomide, and Dexamethasone (IsaPd) in Daratumumab‐Refractory Multiple Myeloma Patients: A Multicenter Real‐World Study
Source: Hematol Oncol. 2025 Feb 3;43(2):e70042. doi: 10.1002/hon.70042 (PMC11789454; doi:10.1002/hon.70042)

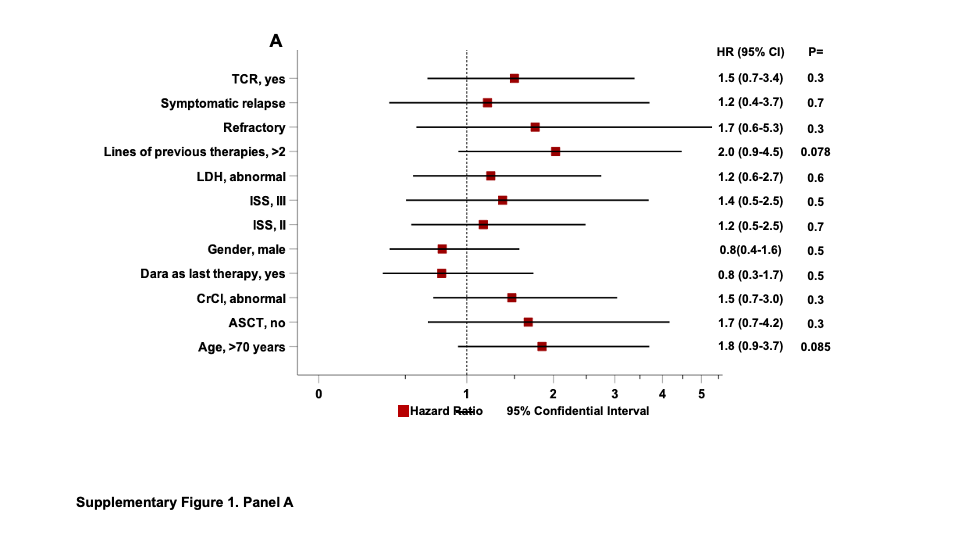

Supplement: Supplementary file 2 — Figure S1A [file HON-43-e70042-s001.tiff]

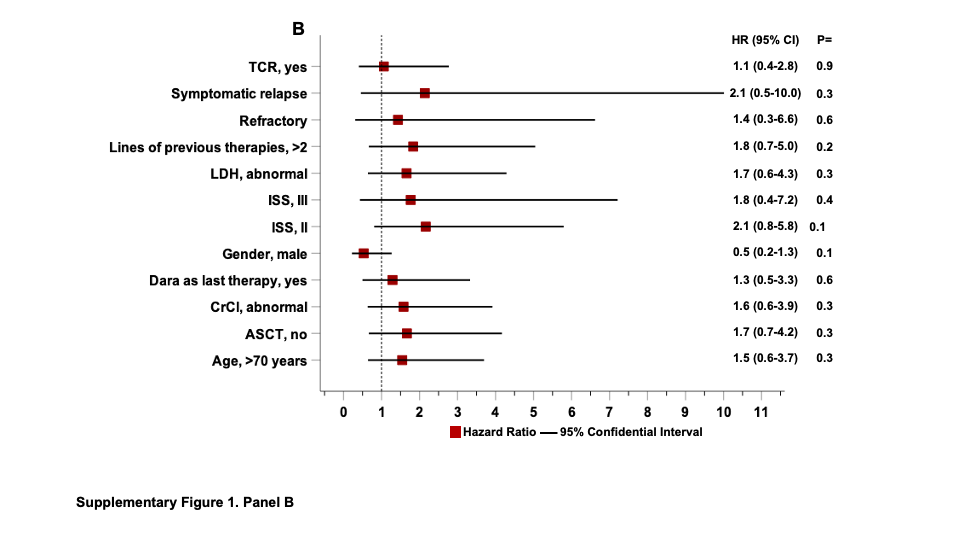

Supplement: Supplementary file 3 — Figure S1B [file HON-43-e70042-s005.tiff]

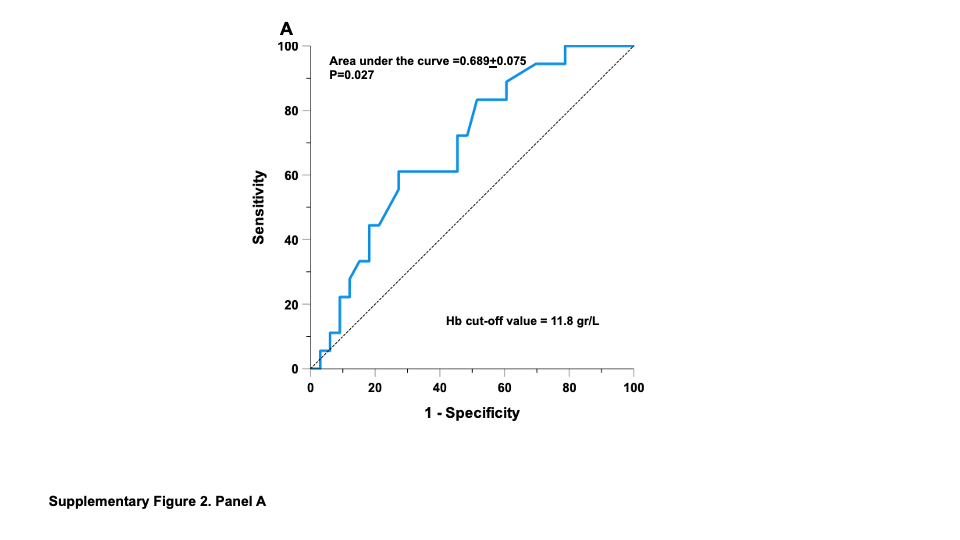

Supplement: Supplementary file 4 — Figure S2A [file HON-43-e70042-s002.tiff]

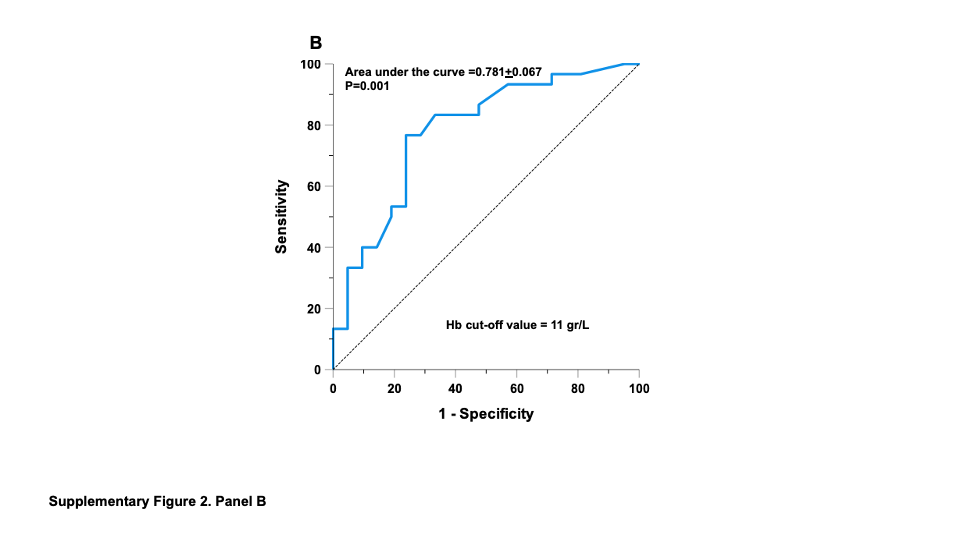

Supplement: Supplementary file 5 — Figure S2B [file HON-43-e70042-s004.tiff]
